# A Multi-Site Knowledge Attitude and Practice Survey of Ebola Virus Disease in Nigeria

**DOI:** 10.1371/journal.pone.0135955

**Published:** 2015-08-28

**Authors:** Garba Iliyasu, Dimie Ogoina, Akan A. Otu, Farouq M. Dayyab, Bassey Ebenso, Daniel Otokpa, Stella Rotifa, Wisdom T. Olomo, Abdulrazaq G. Habib

**Affiliations:** 1 Infectious and Tropical Diseases Unit, College of Health Sciences, Bayero University, Kano, Kano State, Nigeria; 2 Department of Internal Medicine, Niger Delta University/Niger Delta University Teaching Hospital (NDUTH), Okolobiri, Bayelsa State, Nigeria; 3 Department of Internal Medicine, University of Calabar, Calabar, Cross River State, Nigeria; 4 Infectious and Tropical Diseases Unit, Department of Medicine, Aminu Kano Teaching Hospital, Kano, Kano State, Nigeria; 5 Nuffield Centre for International Health and Development, University of Leeds, Leeds, United Kingdom; 6 Department of Public Health, Federal Medical Center, Yenagoa, Bayelsa State, Nigeria; 7 Infection Control Unit, Niger Delta University Teaching Hospital, Okolobiri Bayelsa State, Nigeria; Georgia State University, UNITED STATES

## Abstract

**Background:**

The 2014 Ebola Virus Disease (EVD) outbreak was characterised by fear, misconceptions and irrational behaviours. We conducted a knowledge attitude and practice survey of EVD in Nigeria to inform implementation of effective control measures.

**Methods:**

Between July 30^th^ and September 30^th^ 2014, we undertook a cross sectional study on knowledge, attitude and practice (KAP) of Ebola Virus Disease (EVD) among adults of the general population and healthcare workers (HCW) in three states of Nigeria, namely Bayelsa, Cross River and Kano states. Demographic information and data on KAP were obtained using a self-administered standardized questionnaire. The percentage KAP scores were categorised as good and poor. Independent predictors of good knowledge of EVD were ascertained using a binary logistic regression model.

**Results:**

Out of 1035 study participants with median age of 32 years, 648 (62.6%) were males, 846 (81.7%) had tertiary education and 441 (42.6%) were HCW. There were 218, 239 and 578 respondents from Bayelsa, Cross River and Kano states respectively. The overall median percentage KAP scores and interquartile ranges (IQR) were 79.46% (15.07%), 95.0% (33.33%) and 49.95% (37.50%) respectively. Out of the 1035 respondents, 470 (45.4%), 544(52.56%) and 252 (24.35%) had good KAP of EVD defined using 80%, 90% and 70% score cut-offs respectively. Independent predictors of good knowledge of EVD were being a HCW (Odds Ratio-OR-2.89, 95% Confidence interval-CI of 1.41–5.90), reporting ‘moderate to high fear of EVD’ (OR-2.15, 95% CI-(1.47–3.13) and ‘willingness to modify habit’ (OR-1.68, 95% CI-1.23–2.30).

**Conclusion:**

Our results reveal suboptimal EVD-related knowledge, attitude and practice among adults in Nigeria. To effectively control future outbreaks of EVD in Nigeria, there is a need to implement public sensitization programmes that improve understanding of EVD and address EVD-related myths and misconceptions, especially among the general population.

## Introduction

Ebola Virus Disease (EVD) also referred to as Ebola haemorrhagic fever is a highly lethal haemorrhagic disease named after a river in the Democratic Republic of the Congo (formerly Zaire) where it was first described in 1976 [1]. It is caused by a virus of the family Filoviridae, genus Ebola virus. The genus Ebola virus is divided into five different species (the Zaire, Sudan, Tai Forest, Bundibugyo, and Reston viruses), which differ in their virulence for humans. Its natural reservoirs are thought to include fruit bats and non-human primates. Since its discovery, over 20 confirmed outbreaks have been reported [1, 2]. EVD is transmitted by physical contact with body fluids, secretions, breast milk, tissues or semen from infected persons that are alive or immediately following death especially during funeral rites. Transmission from patients within healthcare settings has followed a typical pattern as patients are often treated by unsuspecting healthcare personnel without the appropriate protective equipment. The typical natural history of the disease begins with an average incubation period of 2–21 days [2,3,4]. Patients present most frequently with initial flu-like symptoms, fever, asthenia, diarrhea, abdominal pain, headache, joint aches, muscle aches, sore throat and conjunctivitis [2,3,4]. Infected individuals receive limited care as no specific treatment or vaccine exists, and they typically die within few variable number of days of their initial infection with 50–90% case fatality rate (CFR) [5,6]. There is a marked variation in clinical course of EVD across the different viral species with Zaire Ebola virus being the most lethal and causing CFR of up to 90% [4,7].

**Fig 1:**
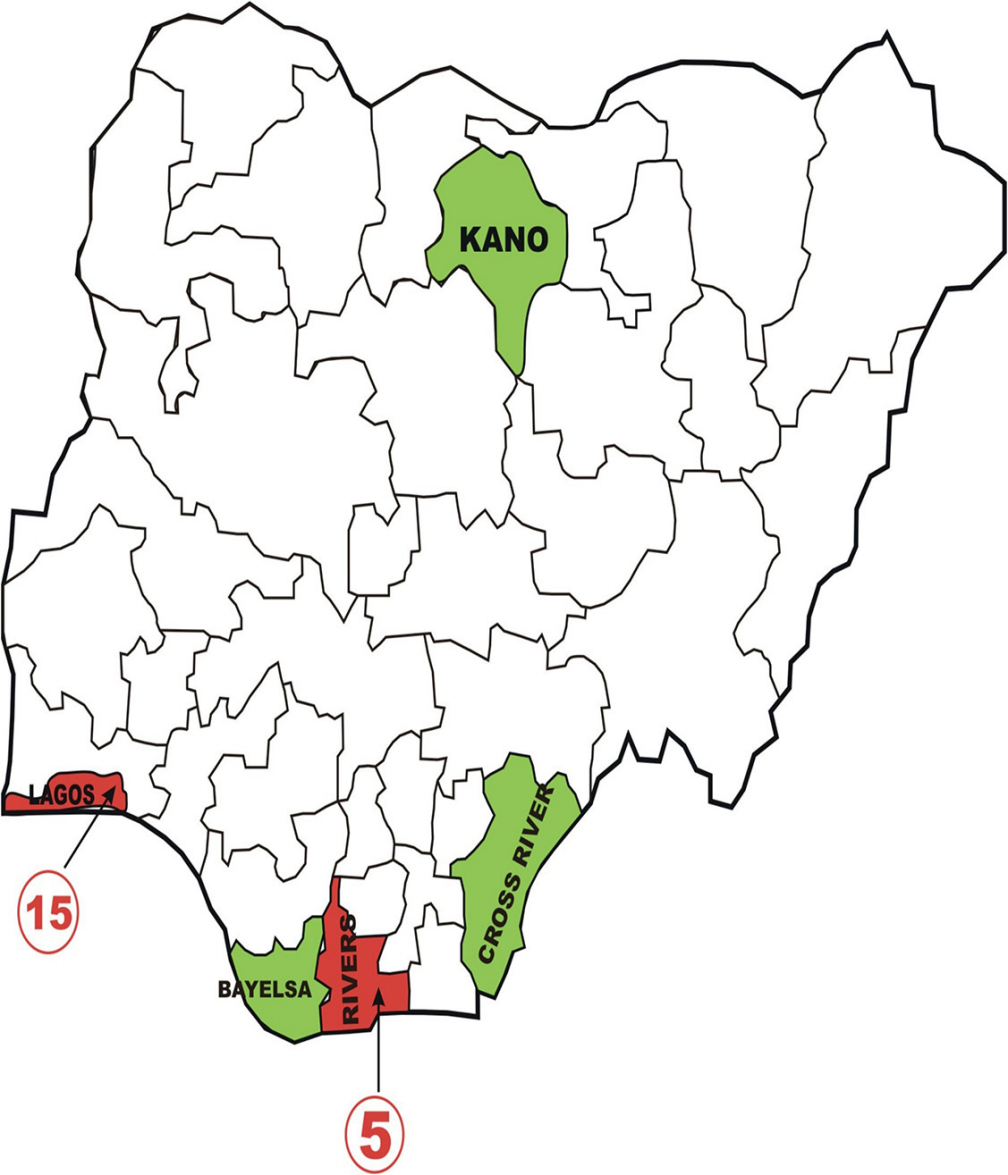
Map of Nigeria showing states with Ebola reported cases (red) and sites of survey (green).

The current outbreak of EVD that started in December 2013 in Guinea with reported cases first notified in March 2014 has defied several months of mitigation and containment efforts [8]. As at 14^th^ June 2015, there were 27,341 suspected EVD cases with 11,184 fatalities (41%) reported mainly from Guinea, Liberia and Sierra Leone [9]. The disease has affected other countries in the sub-region to a lesser extent notably Mali, Nigeria and Senegal. Countries outside of Africa have also recorded EVD cases arising from the current outbreak and they incude Italy, Spain, United Kingdom and the United States of America [9]. Gene sequencing of the virus causing the 2014 West African outbreak confirmed it to be indistinct from the Ebola-Zaire virus with 98% homology but a slightly lower CFR [2]. On 20^th^ July 2014, the outbreak reached Nigeria through an infected diplomat who flew to Lagos from Liberia. Following recognition of the index case and declaration of an outbreak on 24^th^ July by Nigerian authorities, there was widespread panic and countrywide dissemination of conflicting and potentially harmful information on the social media [10, 11, 12]. At least two lives were lost following drinking of salty water, rumored to be protective against EVD [13]. By the end of the outbreak in late September 2014, a total of 20 confirmed Ebola cases, 891 contacts and eight deaths, had been reported in Lagos and Rivers states of the country (Fig 1) [11,12,14,15]. After concerted efforts by national and international agencies, EVD in Nigeria was successfully contained and Nigeria was declared EVD free by the World Health Organization (WHO) on 20^th^ October 2014

The emergence and spread of EVD was accompanied by fear, misconceptions and unusual behaviours by the general population and by health workers within and beyond affected countries and continents [10,13,16]. In view of the unprecedented spread of the EVD and its significant negative consequences on the health and socioeconomic life of communities, countries and regions, the WHO had on 8^th^ August 2014 declared EVD outbreak a Public Health Emergency of International Concern (PHEIC) [17]. The WHO had emphasized the need to improve EVD-related health information to dispel misconceptions and myths. The adopted strategies would mitigate fear, prevent stigma and discrimination, and ultimately halt the current and future outbreaks of EVD. To determine the awareness level and perceptions related to EVD in Nigeria, we aimed to ascertain the knowledge, attitude and practice of EVD in three states of Nigeria. We hope our findings will provide necessary information that might guide the development and implementation of public health strategies to prevent and control EVD in the sub-region.

## Materials and Methods

### Ethics approvals

Ethics approval was obtained from the respective ethical committees of the tertiary hospitals: AKTH, Kano (Approval reference AKTH/MAC/SUB/12A/P-3/VI/1372), UCTH, Calabar (Approval reference UCTH/HREC/33/192), FMC Yenagoa and NDUTH, Okolobiri (Approval reference NDUTH/REC/054/2014). These committees were certified by the Nigerian National Health Research Ethics Committee as contained in their database (http://nhrec.net/nhrec/registered-health-research-ethics-committees-in-nigeria-hrec/). The responses of study participants were treated confidentially. All potential participants were provided information about the survey and only those who gave written consent were included in the study.

### Study design

This was a cross sectional analytical study undertaken between 30^th^ July and 30th September 2015 among the general population and healthcare workers of three states in Nigeria, including Kano, Bayelsa and Cross Rivers States (Fig 1).

### Study setting and sites

It was undertaken among the general population including participants in higher institutions in Kano city, Kano state and among healthcare workers of four tertiary hospitals in Kano, Bayelsa and Cross River States. The tertiary hospitals were Aminu Kano Teaching Hospital (AKTH) in Kano state, Niger Delta University Teaching Hospital (NDUTH) and Federal Medical Center (FMC), Yenagoa both in Bayelsa state and University of Calabar Teaching Hospital (UCTH) in Cross River state.

Kano is an urban metropolis located in the North-West zone of Nigeria and has the AKTH, a 550 bed hospital that serves as a major referral facility in the zone. The hospital served as the coordinating center of the study as well as a study site.

Bayelsa state is situated in the South-South zone of Nigeria. The NDUTH is a 200 bed State Government-owned tertiary hospital situated in Okolobiri, a semi-urban city in Bayelsa state while the FMC is a 400 bed Federal Government of Nigeria owned tertiary hospital situated in Yenagoa, the capital city of Bayelsa state.

The UCTH is the only tertiary health facility in Cross River State which is in the South-South zone of Nigeria. Located in the capital city of Calabar, the UCTH has 1000 beds and provides specialist care for residents of Cross River and people from neighboring states.

### Study participants

We studied the general adult population in Kano metropolis including participants in higher institutions of learning and healthcare workers in the four tertiary hospitals stated above. Study participants were recruited by convenience sampling.

### Data collection

Data was collected from respondents using a standardized self-administered questionnaire (S1 Appendix), distributed as hard copies by trained research assistants. Research assistants explained the purpose of the study to respondents and obtained written consent for the questionnaire to be filled anonymously and returned within an hour or when not possible, at the end of the working day. The questionnaire was pre-tested on a random sample of 10 persons in each tertiary hospital to ensure practicability and validity of questions and interpretation of responses. Following pre-testing, some questions and responses were revised for clarity or deleted as appropriate.

The questionnaire comprised of four parts: a section on demographic and occupational characteristics of study participants; and one section each on knowledge; attitudes; and practice of EVD including open- and closed-ended questions on causation, transmission, risk factors, clinical features as well as prevention and treatment of EVD. Some questions in the sections on KAP were related to misconceptions and myths about EVD.

The questionnaire was developed using information from published literature on EVD, including publications by WHO and the Centers for Disease Control and Prevention (CDC) [1,4,6,7].

### Scoring

Questions with correct and wrong responses were scored 1 and 0 respectively. Where there were grading (trend) of correctness, responses were scored as 0, 1, 2 or higher depending on the options. Questions that were open ended or lacked correct and wrong responses were not scored e.g., demography, source of information, e.t.c.

On fear of EVD, participants were asked to grade their fear of EVD on a scale of 0 to 10, with 0 representing no fear and 10 representing very high levels of fear. Fear of EVD was further categorized/collapsed into two groups at analyses to include ‘little or no fear’ (fear score of 3 or less) and ‘moderate to high fear’ (score of 4 and above). The KAP scores for each study participant were thereafter used to calculate percentage scores out of a possible maximum of 68, 7 and 32 for knowledge, attitude and practice respectively.

The validity of the KAP questionnaire was confirmed by a Cronbach’s alpha internal consistency coefficient of 0.73 for the 3 components.

### Statistical analysis

Data was entered into Microsoft excel and cleaned. The demographic and other baseline characteristics of the study participants were summarized using medians and interquartile ranges (or means and standard deviation) for quantitative variables and proportions for qualitative variables. Percentage KAP scores were presented as median and interquartile ranges. Using the appropriate percentage KAP scores as cut off points based on performance, we categorised the percentage KAP scores into two groups including good KAP and poor KAP. Differences in study variables according to good or poor knowledge of EVD were explored using student’s t-test for quantitative variables while chi-square of fisher’s exact test was used for categorical variables. Predictors of good versus poor knowledge were determined using crude (univariate) measures of effect (Odds Ratio [95% Confidence Interval]). Covariates associated with good knowledge at 20% were imputed in a Logistic Regression model to determine independent predictors of good knowledge of EVD. The model was constructed using backward stepwise approach using Likelihood Ratio Test (LRT) for covariate selection. Pearson’s coefficient of correlations and multivariable linear regression (MLR) were used to determine relationship between knowledge, attitude and practice (behaviour) scores. Level of significance (α) was set at 0.05. Analyses was done using Stata version 11.0 (Texas, USA).

## Results

### Study population and demographic characteristics

A total of 1322 questionnaires were distributed, out of which 287 were excluded because of non-response (108) or missing data (179). Consequently, 1035 participants were studied including 578 (55.8%), 218(21.1%) and 239(23.1%) from Kano, Bayelsa and Cross River states respectively. The characteristics of study participants according to study site are summarized in Table 1.

**Table 1:** Demographic characteristics of study participants.

Demographic variables	Study sites		
	Kano	Bayelsa	Cross-River
	n = 578(55.8%)	n = 218(21.1%)	n = 239(23.1%)
Demographic variables			
Age [years], mean±sd	29.47±9.3	†36.39±9.26	†38.78±8.75
Gender, n (%) Male/Female	398(68.86)/	*104(47.71)/	**146(61.09)/
	180(31.14)	114(52.29)	93(38.91)
Marital status,n(%)Single/Ever Married	297(51.38)/	*69(31.65)/	*163(68.20/
	281(48.62)	149(68.35)	76(31.80)
Education, n(%)Tertiary/Non-tertiary	405(70.07)/	*218(100)/	*223(93.31)/
	173(29.93)	0(0)	16(6.69)
Occupation,n(%)Health Care Workers	29(5.02)/	*189(86.70)/	*223(93.31)/
(HCW)/Non-HCW	549(94.98)	29(13.30)	16(6.69)

*Statistically significant difference at p<0.0001 in chi-square or fisher’s exact test compared to Kano

**Statistically significant difference at p<0.05 in chi-square test compared to Kano

†Statistically significant difference at p<0.0001 in student’s t- test compared to Kano

sd-standard deviation

Out of 1035 study participants, 648 (62.6%) were males, 529 (51.1%) were single (or never married), 846(81.7%) had tertiary education and 441 (42.6%) were healthcare workers (HCW). The studied population had a median age of 32 years (range 15–69 years). There were 62 (6.0%), 169 (16.3%), 166 (16.0%), 254 (24.5%), 129 (12.5%) and 255 (24.6%) respondents in the 15–19 years, 20–24 years, 25–29 years, 30–34 years, 35–39 years and 40 years or older age categories respectively.

Respondents were from several areas in Kano and comprised the least proportion of HCWs (5%). In Bayelsa state, they were drawn from NDUTH (78.9%) and FMC Yenagoa (21.1%). Data was collected from UCTH Calabar in Cross River state. Respondents from Bayelsa and Cross River states were predominantly HCWs.

### Knowledge, Attitude and Practice (KAP) of EVD

Of 880 respondents, 440 (50.0%) correctly defined Ebola as an infection caused by a virus. Percentages of scores for knowledge, attitude and practice were obtained. Based on the distribution of respondent scores, cut-off scores for Knowledge 80%, Attitude 90% and Practice 70%, were used to classify Knowledge, Attitude and Practice with respect to EVD as good or poor. The mean KAP scores and the proportion of respondents with good KAP scores are shown in Table 2.

**Table 2:** Knowledge, Attitude and Practice Percentage Scores by Study Sites.

KAP scores	Study sites		
	Kano n = 578(55.8%)	Bayelsa n = 218(21.1%)	Calabar n = 239(23.1%)
Mean knowledge score ± sd	76.47±11.39(19.18-	71.92±10.16(23.63-	85.94±13.10(13.32-
%(range)	93.16)	98.17)	99.9)
*Proportion with good	261(45.2)	31(14.2)	178(74.5)
Knowledge n(%)			
Mean attitude score ± sd	90.54±17.66(0-	54.59±49.90(0–100)	74.18±14.85(28.57–
%(range)	99.99)		99.99)
†Proportion with good	414(71.6)	119(54.6)	11(4.6)
attitude n(%)			
Mean practice score ± sd	53.48 ± 24.10(0–100)	33.15 ± 14.26(0–81.81)	64.25± 22.90(0–
%(range)	100)		99.90)
‡ Proportion with good practice	143(24.7)	3(1.4)	106(44.4)
n(%)			

* Knowledge score cut-off of ≥80% used to define good knowledge

† Attitude score cut-off of **≥**90% used to define good attitude

‡ Practice score cut-off of ≥70% used to define good practice

sd-standard deviation

The differences in study variables in relation to level of knowledge of EVD (poor and good knowledge) are presented in Table 3.

**Table 3:** Comparison of study variables in relation to level of knowledge of EVD.

Characteristic	Good knowledge	Poor knowledge	Test statistics	P value
	n = 470(45.4%)	n = 565(54.6%)		
Age [years] (mean±SD)	33.5±9.6	32.7±10.6	T = -1.19	0.234
Gender [Males] n(%)	289(61.5)	359(63.5)	χ^2^ = 0.461	0.497
Marital-Status [Singles] n(%)	270(57.5)	259(45.8)	χ^2^ = 13.5	0.0002
Tertiary education n(%)	397(84.5)	449(79.5)	χ^2^ = 4.30	0.0382
HCW n(%)	219(46.6)	222(39.3)	χ^2^ = 5.60	0.018
State:n(%)				
Kano	261(55.5)	317(56.1)	χ^2^ = 167.02	<0.0001
Bayelsa	31(6.6)	187(33.1)		
Cross River	178(37.9)	61(10.8)		
Moderate to high fear for EVD n(%)	344/404(85.2)	359/518(69.3)	χ^2^ = 31.46	<0.0001

Most of the respondents; 465/509(91.4%), 205/208(98.6%) and 238/238(100%) from Kano, Bayelsa and Calabar respectively identified contact with patients secretion as a means of transmitting the infection (Table 4).

**Table 4:** Some KAP responses from the 3 sites.

	Kano	Bayelsa	Calabar
Knowledge			
Natural causative agent of Ebola	162/577(28.0%)	47/66(71.2%)	231/237(97.5%)
Breathing infected air	297/570(42.1%)	6/20(30.0%)	28/239(11.7%)
Secretions	465/509(91.4%)	205/208(98.6%)	238/238(100%)
Bleeding	515/574(89.7%)	204/207(98.6%)	214/235(91.1%)
Wash and disinfect surfaces	487/571(85.3%)	193/204(94.6%)	204/235(86.8%)
Attitude			
Rate your fear for Ebola	450/575(78.3%)	119/184(64.7%)	134/163(82.2%)
Rate your family fear of Ebola	474/573(82.7%)	119/184(64.7%)	165/173(95.4%)
Behaviour			
Willing to modify habit	225/511(44.0%)	54/188(28.7%)	134/231(58.0%)
How often do you wash and disinfect surfaces/utensils	520/564(92.2%)	78/202(38.6%)	209/235(88.9%)
Information source(mass media)	376/573(65.6%)	171/218(78.4%)	122/156(78.2%)

Only 7.1% and 9.7% of the respondent from Bayelsa disagreed with drinking salt water and eating bitter kola respectively as a means of treatment, compared to 87.3% and 77.7% of the respondents from Kano respectively (Table 5).

**Table 5:** Some Knowledge responses from Kano and Bayelsa.

	Kano	Bayelsa
*Risk of acquiring Ebola virus {n(%)}*		
Environment to human transmission	400/549 (72.9)	153/197(77.7)
Contact with bat	500/568(88.0)	198/207(95.7)
Contact with bush meat	493/570(86.5)	197/200(98.5)
Attending funerals	348/552(63.0)	201/207(97.1)
Travel to epidemic area	429/566(75.8)	131/198(66.2)
*Risk group for EVD {n(%)}*		
Hunters	523/577(90.6)	186/199(93.5)
Veterinarians	492/572(86.0)	158/194(81.4)
HCW	541/575(94.1)	209/211(99.1)
*EVD symptoms {n(%)}*		
Fever	563/577(97.6)	209/210(99.5)
Headache	538/574(93.7)	194/203(95.6)
Vomiting	556/578(96.2)	205/210(97.6)
Diarrhea	550/577(95.3)	207/209(99.0)
Weakness	534/574(93.0)	192/198(97.0)
Abdominal pain	480/567(84.7)	167/193(86.5)
*Treatment of EVD {n(%)}*		
Supportive care	234/571(41.0)	140/194(72.2)
Drinking salt water (disagreed)	502/575(87.3)	15/211(7.1)
Eating bitter kola(disagreed)	443/570(77.7)	41/208(9.7)
Do nothing and pray	324/569(56.9)	0/212(0.0)

Most of the respondents from Calabar said they had heard of (94.0%) and read about (81.3%) EVD, while the entire respondents 227/227(100%) agreed with use of face mask, gloves, outer garments, boots and eye protection as a means of protection. Majority 156/231(67.5%) of these respondents knew that; the only treatment for EVD is supportive care, while 234/571(41.0%) and 140/194(72.2%) of those from Kano and Bayelsa respectively knew there is no treatment for EVD.

Majority of the respondents from Calabar 169/235(71.9%) said they were not willing to work in a unit caring for EVD patients. Of 218 respondents in Cross River state, 19 (8.7%) had never used outer garment, gloves, face mask, boot or eye protection. Most of the respondents from Kano 552/576(95.8%) and Calabar 235/239(99.2%) identified EVD as a serious disease.

Healthcare workers: There were 441 respondents who were healthcare workers with 29 (males 21), 189 (males 88) and 223 (males 135) from Kano, Bayelsa and Cross River states respectively. Their median ages were 33, 34 and 38 years from the three respective states. Among healthcare workers the proportion with good knowledge was 22 (75.9%), 27 (14.3%) and 170 (76.2%) from Kano, Bayelsa and Cross River states respectively. Their respective proportions with good attitude were 22 (75.9%), 105 (55.6%) and 11 (4.9%). While the proportions with good behavior were 12 (41.4%), 3 (1.6%) and 102 (45.7%) from Kano, Bayelsa and Cross River states respectively.

Similarly, the proportion of participants with good knowledge was 219/441 (49.7%) among healthcare workers compared to non-healthcare workers 251/594 (42.3%) (χ^2^ = 5.60; p = 0.018) while the proportion with good behavior was 117/441 (26.5%) compared to 135/594 (22.7%) respectively (χ^2^ = 1.99; p = 0.159). However, the proportion of healthcare workers with good attitude was 138/441 (31.3%) compared to 406/594 (68.4%) among non-healthcare workers (χ^2^ = 139.4; p < 0.0001).

In a logistic regression model, the independent predictors of good knowledge of EVD were being a HCW, reporting ‘moderate to high fear of EVD’ and ‘willingness to modify habit’ pre-Ebola epidemic habits (Table 6). Being single and having tertiary education were statistically significant in a univariate analysis, but not in the logistic regression model.

**Table 6:** Predictors of Good Knowledge of EVD in Multivariate analysis (Logistic Regression); Log Likelihood Ratio -472.06, R^2^ = 17.36.

		Crude Odds Ratio [95%CI]	Adjusted Odds Ratio [95%CI]
Marital Status	Ever Married	1.0 (reference)	1.0 (reference)
	Single	1.60 (1.24–2.05)	1.36 (0.94–1.97)
Age Groups [15-19y; 20-24y; 25-29y; 30-34y; 35-39y; ≥40y]	15–19 years	1.0 (reference)	1.0 (reference)
	For each Increasing Age Group	1.02 (1.00–1.04)	1.01 (0.99–1.04)
Educational Level	Non-Tertiary	1.0 (reference)	1.0 (reference)
	Tertiary	1.41 (1.02–1.94)	1.38 (0.91–2.08)
Occupation	Non-Health Care Worker	1.0 (reference)	1.0 (reference)
	Health Care Worker	1.35 (1.05–1.73)	2.89 (1.41–5.90)
Fear of EVD	Little or no fear	1.0 (reference)	1.0 (reference)
	Moderate to high fear	2.54(1.81–3.56)	2.15 (1.47–3.13)
Willingness to modify habit	Not willing	1.0 (reference)	1.0 (reference)
	Willing	2.32(1.76–3.04)	1.68(1.23–2.30)
State	Kano	1.0	1.0
	Bayelsa	0.20(0.13–0.31)	0.06(0.03–0.14)
	Cross River	3.54(2.51–5.03)	1.12(0.52–2.41)

There was weak positive correlation between overall percent knowledge score and overall percent attitude score (r = 0.1366; p<0.001). However, there was a slightly better positive correlation between overall percent knowledge score and overall percent behaviour score (r = 0.2757; p<0.0001). In MLR, there was a significant positive relationship between appropriate behaviour (practice) score to knowledge score, attitude score and to being single (Table 7).

**Table 7:** Multiple linear regression model of Appropriate Behaviour Score; F (6,1028) = 18.16, p< 0.0001, R^2^ 9.58%, adjusted R^2^ 16.6%.

Variable	ß coefficient	T	p-value
Occupation: HCW vs non-HCW	-1.325	-0.72	0.473
Marital status: Single vs Ever-married	3.85	2.38	0.017
Age group	0.16	1.43	0.153
Educational level: Tertiary vs non-tertiary	-2.16	-1.04	0.298
% appropriate knowledge score	0.50	8.15	<0.0001
% appropriate attitude score	0.08	3.26	0.001
Constant	-1.89		

## Discussion

Currently, no approved antiviral drug or vaccine exists against EVD although there are various experimental treatments and vaccines at various stages of testing for safety and effectiveness. Therefore, the only options to curtail epidemics are to prevent transmission of infection by implementing effective containment measures in the community and healthcare settings. To achieve this, better understanding of people’s knowledge, attitude and practices (KAP) toward EVD is needed.

Apart from an unpublished descriptive technical report from Sierra Leone [18], to the best of our knowledge, this is the first comprehensive analytical KAP survey on EVD in sub Saharan Africa. Our survey confirms significant gaps in knowledge, attitude and practices of EVD among predominantly educated participants with over 80% of them having tertiary education and over 40% being HCWs. The deficiencies in KAP were observed in all the three study states in Nigeria.

For instance, only half of 880 respondents in our study knew the causative agent of EVD, with an even lower response among respondents in Kano compared to Bayelsa and Cross River State. These differences across the states may be attributed to the higher proportion of HCW in Bayelsa and Cross River states. Nonetheless majority of respondents correctly associated EVD with bats, monkeys and wild animals; recognized it as a very serious and fearful disease; and correctly identified its basic symptoms. Furthermore, a majority of respondents were aware of the means of transmission of EVD, albeit fewer respondents from Kano (a predominantly Muslim community) were aware of the risk of transmission during funerals. Similarly, respondents’ overall knowledge of the means of personal prevention such as hand washing with soap and water, using facemask, eye protection, outer garment and boots was good. In spite of good knowledge of personal protection, very few people were willing to modify their habits.

Our study identified differences in knowledge about the treatment of EVD between the 3 sites perhaps partly influenced by the differing characteristics of the respondents in Kano, Bayelsa and Calabar. For example, it is understandable that most respondents from Bayelsa and Calabar (who are predominantly HCWs) recognized supportive care as the most appropriate means of management compared to very few people in Kano (with the least proportion of HCWs). Interestingly, most respondents from Bayelsa were uncertain about the value of drinking salt water or eating bitter kola for treating EVD, a situation which coincided with the proliferation of myths and phony cures for EVD on social media sites such as Facebook and Twitter [10, 19]. Evidence reveals that myths and harmful misconceptions are not limited to Nigeria alone [16], as a recent survey in Sierra Leone indicated that 2 in 5 respondents believed that bathing with salt and hot water could protect them from Ebola [18]. The popularity of traditional beliefs or remedies such as wearing dry banana leaves to protect against epidemics of infectious diseases has also been reported in Uganda [20]. It is possible that awareness campaigns implemented in Bayelsa during the EVD epidemic in Nigeria did not sufficiently dispel the misconceptions which were propagated via social media. While the proliferation of computers, tablets, and smartphones in the West African sub-region provide channels for the rapid dissemination of information through the internet and social media, the risk of blending facts with misinformation via these largely unregulated platforms is ever present [19]. Misinformation arising from such sources may potentially confer on the populace a false sense of protection whilst promoting resistance towards or even eroding confidence in proven preventive measures. Consequently, public health and government agencies should promptly and vigorously address such misinformation with correct health information and education [19].

This study identified the predictors of ‘good knowledge’ as: being a HCW, having ‘moderate to high fear for EVD’; and a ‘willingness to modify old habits’. Consistent with this, there were generally good attitudes and behaviour towards key means of preventing EVD with exception of respondents from Bayelsa. The study showed that, overall appropriate or ‘good knowledge’ was low (45%) and correlated poorly with attitude. Indeed, willingness to modify habits was generally low in all the three states (44–58%). The reasons are not clear however it is instructive that good knowledge of EVD was significantly higher among HCWs but the good attitude was significantly higher among the non-HCWs when compared with HCWs. Over 70% of HCWs from Calabar were unwilling to work in units caring for EVD. The reservations expressed by these workers about caring for EVD patients may stem from a fear of contracting EVD which has high mortality and no known cure. The lack of personal protective equipment (PPE) in their clinical settings may also be contributing to this reluctance among HCWs. Indeed, approximately 869 African HCWs have been infected and 507 (58%) have died since the beginning of the West African EVD epidemic [9]. A similar attitude was observed in Sierra Leone, among HCWs with poor access to PPE [18]. Our finding of poor attitude towards EVD among HCWs is undesirable and may be addressed by provision of PPE while intensifying education on infection prevention and control practices in healthcare settings. Governments should also explore provision of insurance policy, job security, incentives to motivate work and provision for families of HCW should the worst happen. These strategies may inspire greater confidence among HCWs in their ability to remain healthy while caring for such highly infectious patients.

Notwithstanding codes of ethics requiring health professionals not to abandon sick patients, Nancy Kass [21] points out that helping patients with an illness that is both highly contagious and usually fatal is “supererogatory', i.e., it goes above and beyond usual norms of good ethical conduct. However our study also showed that respondents with ‘good knowledge’ were more likely to have ‘good behaviour’. Thus, improving knowledge can potentially improve behaviour and possibly attitude. Although our survey found non-HCWs were less knowledgeable about Ebola, interventions aimed at addressing critical knowledge gaps should target all sectors of the population regardless of age, education and occupation. These educational packages will need to be tailored to suit the perculiarities of various groups such as infection prevention and control education for HCWs.

Despite recognition of funeral practices as a major source of EVD transmission [22], a surprising finding was that only 63% of respondents from Kano correctly identified attending funerals as a source of EVD transmission compared to 97.1% among mainly HCWs from Bayelsa. Furthermore only 10.1% of respondents from Kano reported reducing physical contact with others to less than 10 times during the day following the EVD outbreak in Nigeria. This is a concern especially as transmission may be facilitated in largely Muslim settings where funerals are hurriedly conducted immediately following a person’s death. In a similar but smaller unpublished survey in Sierra Leone, International Red Cross reported that only 26.7% of respondents in Kailahun and 21.4% in Kenema regions of sierra Leon knew that avoiding the dead remains of an infected individual could prevent transmission of EVD. These findings all support the need for greater communication and engagement of communities and local leaders in the promotion of safe burial rites during EVD outbreaks.

The overall level of knowledge on EVD in the Nigeria study is comparable to findings in the unpublished KAP survey in Sierra Leone [18] even though the latter was conducted among the general population in contrast to our study conducted among both HCWs and non-HCWs. In both studies, nearly one third of the respondents believed EVD is transmitted by air droplets. It is also well documented that HCWs seldom have the best knowledge or attitude concerning emerging infectious diseases. For example, several KAP studies on viral haemorrhagic fevers indicate that HCWs have low knowledge and poor attitude [23, 24, 25, 26, 27] as observed among respondents in this survey.

The proportion of respondents in our study, with positive change in behaviour such as hand washing with soap and water was lower than reported in the Sierra Leone survey [18]; however the proportions of respondents who reported reducing physical contact were comparable and generally low. These findings highlight the inherent difficulty in changing culturally entrenched behaviours such as handshakes and hugs as forms of greeting in African communities.

Although the sample size obtained from multiple sites across Nigeria adds to the survey’s strengths, this study has some limitations. Firstly, though the survey was conducted about the same time (July-September 2014) in all 3 states, there were slight differences in actual duration and period of data collection in each site sites leading to variability in responses as global health information about EVD changed rapidly and often dramatically. Secondly, though the study provides essential insight into gaps in knowledge, attitude and practice, the findings should be generalized with caution as the study population was comprised mainly those with tertiary education and HCWs within the Nigerian sociocultural setting. Furthermore the study sites had no affected EVD cases and findings represent responses from just three states which might not be representative of the whole country. Thirdly, slight differences in the nature of the data between the sites precluded the analysis of some data from Cross River State. Lastly, the questionnaire was comprehensive on knowledge but appeared less comprehensive on aspects pertaining to attitude and practice (behaviour).

In conclusion, we recommend the implementation of massive and sustained public awareness programmes to increase the knowledge of the general populace, focusing on mode of transmission, context-specific preventive measures such as addressing myths and misconceptions; and promoting safe burial practices. Health systems strengthening should be promoted by providing frontline HCWs with PPE and essential information about EVD while highlighting infection control measures to boost the morale and capacity of HCWs to respond to outbreaks of Ebola infection.

## Supporting Information

S1 Appendix (DOC)
