# Supplementary material for: A Multi-Site Knowledge Attitude and Practice Survey of Ebola Virus Disease in Nigeria
Source: PLoS One. 2015 Aug 28;10(8):e0135955. doi: 10.1371/journal.pone.0135955 (PMC4552659; doi:10.1371/journal.pone.0135955)
Supplement: S1 Appendix — (DOC) [file pone.0135955.s001.doc]

**A. DEMOGRAPHIC AND PRACTICE CHARACTERISTICS**

**A1.** How old were you on your last birthday? _________

**A2.** Gender (don’t ask) Male [ ] Female [ ]

**A3.** What is your marital status? Married [ ] Single (never married) [ ] Other……………..

**A4.** How many children do you have? _______________

**A5.** What is your education level? _________________________________________________

**A6.** Where do you occupation/work? ______________________________________________

**A7.** What type of work do you do ?________________________________________________

**B. KNOWLEDGE**

This section of the interview is designed to explore your knowledge related to Ebola Virus Disease.

**B1.** How do you define Ebola? ____________________________________________________________________________________________________________________________________________________________

I will read to you some statements regarding Ebola. For each statement, please give me a yes or no answer.

**B2.** Ebola is transmitted by:

Breathing infected air Yes[ ] No[ ]

Animal-to-human Yes[ ] No [ ]

Animal-to-animal Yes[ ] No[ ]

Human-to-human Yes[ ] No[ ]

Environment-to-human Yes[ ] No[ ]

Eating bush meat Yes[ ] No [ ]

Saliva, nasal secretions, excreta, feces, and fomites of infected person Yes [ ] No [ ]

Contact with Bats Yes [ ] No [ ]

Contact with Bush meat Yes [ ] No [ ]

Contact with Rodents Yes [ ] No [ ]

Attending/participating in funeral of a deceased Ebola patient Yes [ ] No [ ] if yes,

What do you believe may pose a risk during funeral rites? Specify____________________

Travel to places where there is an epidemic Yes [ ] No [ ]

Visiting clinic where Ebola patients are isolated Yes [ ] No [ ]

**B3.** Individuals at risk for contracting Ebola are:

Hunters Yes [ ] No [ ]

Veterinarians Yes [ ] No [ ]

Health workers Yes[ ] No[ ]

Contact with patients Yes[ ] No[ ]

Travel to epidemic areas Yes[ ] No[ ]

**B4.** What are the Symptoms of Ebola virus

{1} Fever Yes[ ] No[ ]

{2} Headache Yes[ ] No[ ]

{3} Vomiting Yes[ ] No[ ]

{4} Diarrhoea Yes[ ] No[ ]

{5} Intense fatique/general weakness Yes[ ] No[ ]

{6} Abdominal Pain Yes[ ] No[ ]

{7} Bleeding Yes[ ] No[ ]

{8} Other – specify____________________________________________________________

I am going to read a list of measures concerning prevention of Ebola and for each of them, I would like you to tell me whether you agree, are uncertain or disagree.

**B5.** Do you know about universal health precautions ? yes[ ] no [ ]

**B6.** Wash hands with soap and water Agree[ ] Uncertain[ ] Disagree [ ]

**B7.** Face mask Agree [ ] Uncertain [ ] Disagree [ ]

**B8.** Gloves Agree [ ] Uncertain [ ] Disagree [ ]

**B9.** Outer garments Agree [ ] Uncertain[ ] Disagree [ ]

**B10.** Boots or boot covers Agree[ ] Uncertain [ ] Disagree [ ]

**B11.** Eye protection Agree [ ] Uncertain [ ] Disagree [ ]

**B12.**Wash and disinfect utensils Agree[ ] Uncertain [ ] Disagree[ ]

**B13.**Wash and disinfect surfaces Agree [ ] Uncertain [ ] Disagree [ ]

**B14.** Is there treatment for Ebola Yes [ ] No [ ], if yes specify……………………………

{3} don’t know [ ]

**B15.** How will the spread of ebola be prevented in the community?

{1} everybody should take salt water bath Agree [ ] Uncertain [ ] Disagree [ ]

{2} drink salt water Agree[ ] Uncertain[ ] Disagree [ ]

{3} eating bitter kola Agree[ ] Uncertain[ ] Disagree [ ]

{4} avoid crowded places if one can help it Agree[ ] Uncertain[ ] Disagree [ ]

{5} use face mask when going to crowded places Agree [ ] Uncertain [ ] Disagree [ ]

{6} minimize or avoid shaking hands with other people Agree[ ] Uncertain[ ]

Disagree [ ]

{7} wash hands after using public conveniences / toilets Agree[ ] Uncertain[ ]

Disagree [ ]

{8} wash hands after shaking other people Agree[ ] Uncertain[ ] Disagree [ ]

{9} use hand sanitizers frequently after shaking people Agree[ ] Uncertain[ ]

Disagree [ ]

{10} avoid travelling to towns with suspected cases or going to areas of suspected

outbreaks Agree[ ] Uncertain[ ] Disagree [ ]

{11}use of personal equipments for haircut, manicure,etc Agree[ ] Uncertain[ ]

Disagree [ ]

{12} no need to do anything continue living as before, God will protect. Agree[ ]

Uncertain[ ] Disagree [ ]

**B16**. Mention where you think Ebola originated from? God’s wrath; Guinea; Liberia; S/Leone; West Africa; Multiple places; Other ________________________________________________

**B17**. Mention from which animal you think Ebola originated from? Bats; Monkeys; Rodents; Others ________________________________________________________________________

**C. ATTITUDES**

I would like to know your attitudes towards Ebola. Try to answer the following questions as truthfully as possible.

**C1.** Ebola may be prevented Agree [ ] Uncertain [ ] Disagree [ ]

**C2.** Ebola is a serious disease Agree [ ] Uncertain [ ] Disagree [ ]

**C3.** How would you rate your fear of getting Ebola on a 1 to 10 scale with 1 meaning no fear at all and 10 very much fear? 1 2 3 4 5 6 7 8 9 10

No fear at all [ ] Very much fear [ ]

**C4.** How would your colleagues/family members rate their fear of getting Ebola on a 1 to 10 scale with 1 meaning no fear at all and 10 meaning very much fear?

1 2 3 4 5 6 7 8 9 10

No fear at all [ ] Very much fear [ ]

**D. BEHAVIORS**

I am going to ask you some questions which are designed to gather information about your behavior.

**D1.** In the past three months have you modified your working habits for fear of getting Ebola?

No [ ]Yes [ ](Specify__________________________________________)

**D2**. How frequently do you have physical bodily contact (handshake etc) in a day pre-Ebola in Nigeria? Number_____/day

**D3**. How frequently do you have physical bodily contact (handshake etc) in a day post-Ebola in Nigeria? Number_____/day

**D3.** In your activity how often do you use each of the following preventive measures to avoid spreading of the Ebola virus (after reading each item ask “Would you say never, rarely, sometimes, often, always?”)?

**D4-1.** Outer garments Always[ ] Often [ ] Sometimes[ ] Rarely[ ] Never [ ]

**D4-2.** Gloves Always [ ] Often [ ] Sometimes [ ] Rarely [ ] Never [ ]

**D4-3.** Face mask Always[ ] Often [ ] Sometimes [ ] Rarely [ ] Never [ ]

**D4-4.** Eye protection Always [ ] Often [ ] Sometimes [ ] Rarely [ ] Never [ ]

**D4-5.** Boots or boot covers Always[ ] Often [ ] Sometimes [ ] Rarely [ ] Never [ ]

**D5.** How often do you wash your hands?

Always [ ] Often [ ] Sometimes [ ] Rarely [ ] Never[ ] (go to **D7**)

**D6.** How do you wash your hands?With water [ ] With water and soap [ ] With disinfectant [ ]

**D7.** How often do you wash and disinfect surfaces and utensils?

Always [ ] Often [ ] Sometimes [ ] Rarely [ ] Never [ ]

**E. INFORMATION**

**E1.** From which of the following sources of information do you receive Ebola education? (more than one answer if possible)

None [ ] Mass media [ ] Friend/Family member [ ] Employer [ ] Health professional [ ] Other (specify ____________________________)

**E2.** Do you feel you need more information about Ebola? Yes [ ] No[ ]
